# Supplementary material for: Neurodynamic Treatment Promotes Mechanical Pain Modulation in Sensory Neurons and Nerve Regeneration in Rats
Source: Biomedicines. 2022 May 31;10(6):1296. doi: 10.3390/biomedicines10061296 (PMC9220043; doi:10.3390/biomedicines10061296)
Supplement: Supplementary file 1 [file biomedicines-10-01296-s001.zip › biomedicines-1747449-supplementary.pdf]

Table S1. Baseline values of animal sensory and motor profiles.

	NO NDT	NDT POST	NDT PRE-POST	df	F	η^2_P	*p*
**Sensory Assessment**							
Mechanical pain threshold injured side (g)	20.8 ± 2.04	17.5 ± 4.18	18.3 ± 4.08	2, 15	1.413	0.159	0.274
Mechanical pain threshold not injured side (g)	18.3 ± 4.08	20 ± 8.94	19.2 ± 4.92	2, 15	0.103	0.014	0.902
Touch threshold injured side (g)	15 ± 0	13.3 ± 2.58	15 ± 3.16	2, 15	1	0.118	0.391
Touch threshold not injured side (g)	15 ± 0	13.3 ± 2.58	14.2 ± 2.04	2, 15	1.154	0.133	0.342
Pain behaviour to noxious stimuli	2 ± 0	2 ± 0	2.17 ± 0.408	2, 15	1	0.118	0.391
Number of paw withdrawals on 30 repeated nerve tensioning	1.83 ± 1.84	3.33 ± 3.08	3.67 ± 2.58	2, 15	0.88	0.105	0.435
**Motor Assessment**							
Grissini test speed (s)	66.5 ± 11.8	76.3 ± 12.5	86.3 ± 10.9 *	2, 15	4.247	0.362	0.035
Grissini test number of adjustments	48.3 ± 14.0	41 ± 9.84	48.3 ± 9.81	2, 15	0.83	0.1	0.455
Grasping test (g)	617 ± 39.5	614 ± 94.1	613 ± 87.1	2, 15	0.004	0.0005	0.996
Rope test (s)	57.8 ± 25.2	32.2 ± 13.3	42.3 ± 31.2	2, 15	1.684	0.183	0.219
Animal weight (g)	238 ± 9.56	237 ± 5.88	239 ± 6.43	2, 15	0.079	0.01	0.925

Table S2. ANOVA for repeated measures results from behavioural assays (η^2_P: partial eta squared).

**Sensory Assays**									
	Time			*Treatment*			*Time x Treatment*		
Variable	**F**	*p*	η^2_P	**F**	*p*	η^2_P	**F**	*p*	η^2_P
Mechanical pain threshold injured side (g)	68.971	**0.0000**	0.798	12.357	**0.0000**	0.191	8.918	**0.0000**	0.505
Touch threshold injured side (g)	5.23	**0.0001**	0.23	8.016	**0.0006**	0.132	1.131	0.343	0.114
Mechanical pain threshold not injured side (g)	2.274	0.042	0.115	1.553	0.216	0.029	0.797	0.652	0.084
Touch threshold not injured side (g)	5.481	**0.0001**	0.238	5.547	**0.005**	0.096	0.73	0.72	0.077
Pain behaviour to noxious stimuli	43.333	**0.0000**	0.712	3.9	**0.023**	0.069	5.533	**0.0000**	**0.387**
**Motor Assays**									
Grissini test speed (sec)	2.405	**0.043**	0.12	5.081	**0.008**	0.104	0.637	0.779	0.067
Grissini test number of adjustments	3.0020	**0.015**	0.147	2.817	0.065	0.06	1.651	0.105	0.158
Grasping test (g)	300.04	**0.000**	0.945	0.729	0.485	0.014	0.858	0.591	0.089
Rope test (sec)	1.7638	0.114	0.092	8.3343	**0.0004**	0.137	0.8732	0.576	0.091
Animal weight (g)	23.395	**0.0000**	0.51	1.778	0.181	0.073	0.522	0.720	0.044
	Treatment			*Side*			*Treatment x Side*		
Wet muscle weight (g)	10.309	**0.00039**	0.407	157.012	**0.0000**	0.84	1.023	0.372	0.064

Table S3. Morphological characteristics of DRG explants.

	**CTR IN**	**CTR OUT**	**NDT**	**F [df]**	**95% CI**	***p***
Dmax (μm)	1552 ± 447	1465 ± 368	2200 ± 524	7.92 [2,27]	234.85–1235.14	0.003
Nmax	188 ± 92.1	94.2 ± 89.6	153 ± 69.5	3.15 [2,27]	131.74–243.46	0.06
Sholl Critical Value	148 ± 267	160 ± 137	278 ± 323	0.79 [2,27]	−19.96–314.96	0.46
